# Cost-effectiveness of primary surgical versus primary medical management in the treatment of patients presenting with advanced glaucoma

**DOI:** 10.1136/bjo-2021-320887

**Published:** 2022-07-26

**Authors:** Ashleigh Kernohan, Tara Homer, Hosein Shabaninejad, Anthony J King, Jemma Hudson, Gordon Fernie, Augusto Azuara-Blanco, Jennifer Burr, John M Sparrow, David Garway-Heath, Keith Barton, John Norrie, Graeme Maclennan, Luke Vale

**Affiliations:** 1 Population Health Sciences Institute, Newcastle University, Newcastle upon Tyne, UK; 2 Department of Ophthalmology, Nottingham University Hospital, Nottingham, UK; 3 Health Services Research Unit, University of Aberdeen, Aberdeen, UK; 4 Centre for Healthcare Randomised Trials (CHaRT), Health Services Research Unit, University of Aberdeen, Aberdeen, UK; 5 Centre for Public Health, Queen's University Belfast, Belfast, UK; 6 School of Medicine, University of St Andrews, St Andrews, UK; 7 Bristol Eye Hospital, University Hospitals Bristol NHS Foundation Trust, Bristol, UK; 8 National Institute for Health Research (NIHR) Biomedical Research Centre, Moorfields Eye Hospital NHS Foundation Trust, London, UK; 9 Edinburgh Clinical Trials Unit, Usher Institute, University of Edinburgh, Edinburgh, UK; 10 NIHR Applied Research Collaboration North East and North Cumbria, NIHR, Newcastle, UK

**Keywords:** Glaucoma, Treatment Medical, Treatment Surgery

## Abstract

**Synopsis:**

Advanced glaucoma is associated with sight loss. This within-trial economic evaluation compares medical and surgical management strategies. At 2 years, medication appears more cost-effective though longitudinal outcomes are an important subject in future research.

**Background/aims:**

Open angle glaucoma (OAG) is a progressive optic neuropathy. Approximately 25% of newly diagnosed patients with OAG present with advanced disease in at least one eye. The vision loss associated with OAG can lead to significant impacts on vision, quality of life and health care resources. The Treatment of Advanced Glaucoma Study is a randomised controlled trial comparing the effectiveness of primary surgical and medical management for newly diagnosed advanced patients with OAG. An economic evaluation was carried out to understand the costs and benefits of each strategy.

**Methods:**

A cost utility analysis was carried out from a National Health Service perspective over a 2-year time horizon inclusive of patient costs. The primary outcome was patient health-related quality of life measured by the EQ-5D-5L, Health Utilities Index 3 (HUI3) and Glaucoma Utility Index (GUI). Results were expressed as incremental cost per QALY gained.

**Results:**

Trabeculectomy was associated with higher costs and greater effect, the EQ-5D-5L results have an incremental cost per QALY of £45,456. The likelihood of surgery being cost-effective at a £20, 000, £30,000 and £50,000 QALY threshold is 0%, 12% and 56%, respectively. The results for the HUI3, GUI and inclusion of patient costs do not change the conclusions of the study.

**Conclusion:**

This is the first study to evaluate management strategies for those presenting with advanced glaucoma. At a 2-year time horizon, medication is the more cost-effective approach for managing glaucoma. Future research can focus on the costs and benefits of the treatments over a longer time horizon.

**What is already known on this topic**

- Those who present with advanced glaucoma have a high risk for developing sight loss.
- There are significant resource implications associated with glaucoma management and currently a lack of evidence as to the most cost-effective management strategy for those presenting with advanced glaucoma.

**What this study adds**

- Trabeculectomy was associated with higher costs and slightly greater quality of life outcomes, medication is more likely to be considered cost effective at a 2-year time horizon.
- These results were consistent across different health-related quality of life measurement tools.

**How this study might affect research, practice or policy**

- This study provides evidence as to the costs and benefits of surgical and medical management of advanced glaucoma. Future research can focus on the cost and benefits of different strategies over a longer time horizon.

## Introduction

Open angle glaucoma (OAG) is a progressive pressure-related optic neuropathy and a major cause of blindness in the UK and worldwide.^1 2^ Intraocular pressure (IOP)related damage to the optic nerve results in visual field loss which is progressive if untreated. However, in the early stages, the disease glaucoma is usually asymptomatic until the vision field loss is severe. Glaucoma can affect many aspects of daily living and can have a profound effect on health-related quality of life, more markedly in those with advanced disease.^3^ Those who present with a high IOP or severe visual field defect are most likely to lose sight despite treatment^4^ and advanced presentation is the greatest risk factor for lifetime blindness.^5^ In the UK, it is estimated that about 25% glaucoma suffers have advanced glaucoma in at least one eye at presentation.^6 7^ In addition, there are significant resource implications associated with management of the disease. It has been estimated that the cost of the management of a glaucoma patient is $2746±1560 (US$2015) over three years.^8^ With pressure on eye care services increasing^9^ it becomes essential to understand the management strategies which are both effective for the patient and provide optimal use of healthcare resources.

Current guidelines in the UK recommend those presenting with advanced OAG are offered augmented trabeculectomy,^10^ however this is not widely offered by ophthalmologists because of a lack of evidence supporting this recommendation^11^ The Treatment of Advanced Glaucoma Study (TAGS) is a pragmatic UK-based multicentre randomised controlled trial comparing the effectiveness of primary surgery (trabeculectomy) versus medical management (eye drops) in patients presenting with advanced glaucoma in at least one eye.^12^ Patients who presented with advanced glaucoma (as classified by the Hodapp-Parrish-Anderson (HPA) criteria^13^) were randomised to either a primary surgical or medical arm. The TAGS trial concluded that at 2 years trabeculectomy and medical management had similar quality of life, safety and vision outcomes, but trabeculectomy achieved a significantly lower IOP. As part of TAGS, a within-trial cost-utility analysis (CUA) was carried out to assess the differential effects on quantity and quality of life as well as the resource implications associated with the management of glaucoma.^14^ The aim of this study is to compare the costs and the benefits associated with primary surgical management compared with primary medical management of patients presenting with advanced glaucoma.

## Materials and methods

The TAGS trial was carried out in 27 National Health Service (NHS) secondary care glaucoma departments. Each participating department had at least one fellowship-trained glaucoma specialist. The outcomes were collected over a 24-month period of follow-up.^12^ Study participants had severe glaucomatous field loss (HPA classification) in one or both eyes, were over 18 years old and able to consent to participate. Participants were excluded if they were unable to undergo a trabeculectomy, had a high risk of surgical failure, had secondary glaucoma, were pregnant or trying to conceive. Where patients had both eyes eligible the eye with the least amount of visual field loss was deemed the index eye for analysis of clinical outcomes. The within-trial CUA was carried out from both a health care system alone and including a patient perspective with a time horizon of 2 years.

### Health care costs

Participant resource use, in terms of the type and frequency of resources used, was measured using bespoke case report forms (CRFs) which captured the use of resources such as intervention costs, surgery procedures, medications, post-surgery interventions and management of any resulting complications. The CRF was completed at baseline, 4 months, 12 months and 24 months, An additional specific surgery CRF was completed to capture actual surgery costs. In addition, the CRF asked about secondary healthcare resources utilised including; visits to the ophthalmology outpatient clinic and outpatient procedures which the participants utilised during the trial. Costs in this study were given in GBP (2018). Unit costs for procedures and outpatient visits were derived from National Reference Costs 2017/2018^15^ and the British National Formulary.^16^ In addition to the CRF’s an additional patient questionnaire (PQ) was administered to participants at 4, 12 and 24 months follow-up. The PQ asked participants about primary care services they have accessed, including general practitioner visits, community optometrist visits and community nurse appointments. Unit cost for these were derived from the Unit Costs of Health and Social Care 2018.^17–19^

### Patient costs

In addition to healthcare costs, participants’ out-of-pocket expenses were calculated so that the perspective could be expanded beyond that of the UK NHS. To inform this analysis trial participants completed a time and travel questionnaire. The responses to this questionnaire were used to estimate unit costs to patients and their families of the time and travel cost of accessing each type of healthcare provider. These unit costs were combined with information on the frequency of use of services collected using the CRFs and PQ described above.

With respect to travel unit costs, if the journey was undertaken using public transport the fare was used to represent travel costs. If a journey was undertaken by private car, then a fuel rate of £0.45 per mile was applied based on the business and self-employed expenses rate per mile.^20^ The cost of participant time was valued using average costs from the Office of National Statistics.^21^ Paid work, childcare, caring for relative or friend and voluntary work was valued as £13.88 an hour, housework and leisure activities were valued at £10.10 an hour. Time spent in unemployment, retirement or in full time education was valued as £6.04 an hour. Finally, private out of pockets expenses (eg, private appointments, spectacles) as captured on the PQ were included in patient time and travel costs. A table describing each of the unit costs is included in online supplemental tables A1–A5.

10.1136/bjo-2021-320887.supp1 Supplementary data

### Estimation of effects

The impact of treatments on health-related quality of life (HRQoL) were captured using three tools: the EQ-5D-5L, the Health Utilities Index Mark 3 (HUI3) and the Glaucoma Utility Index (GUI).^22–24^ In this base case analysis, the primary outcome was based on the results of the EQ-5D-5L, as this is a measure recommended by National Institute for Health and Care Excellence (NICE) for technology appraisal.^25^ The HUI3 and GUI were investigated as part of additional sensitivity analysis. Responses to the EQ-5D-5L were converted into utilities using the cross-walked values from the EQ-5D-3L dataset^22^ and were used to estimate quality-adjusted life years (QALYs) using the under the curve approach.^26^ The quality of life instruments was administered at seven time points during the course of the trial; baseline, 1, 3, 6, 12, 18 and 24 months post-randomisation. In a sensitivity analysis, QALYs were also estimated using the responses to the HUI3 and GUI instruments. Both costs and outcomes were discounted at a rate of 3.5%.

### Data analysis

The regression analysis chosen for the TAGS within-trial cost-effectiveness analysis was a seemingly unrelated regression (SUREG). A SUREG is a regression model which permits the simultaneous estimation of costs and effects, calculated at an individual level, that could affect both costs and effects and lead to potential correlation between these two dependent variables. There is evidence that a SUREG improves precision surrounding cost-effectiveness estimation in trial-based economic evaluations.^27^ Trial data were used to derive a total NHS cost per participant. A SUREG was used to identify any difference between the surgical and medical arm of the trial while controlling for any modifying factors such the participant’s age and their baseline utility score. This method was also used to determine incremental costs and effects for all sensitivity analyses.

### Sensitivity analysis

To address the robustness of the economic conclusions of the study both stochastic and deterministic sensitivity analyses were undertaken. First, to assess the robustness of the study sampling, non-parametric bootstrapping was carried out. Bootstrapping is a statistical procedure that resamples a single dataset to create many simulated samples to assess statistical precision.^28^ In this study, 1000 iterations of the bootstrapping procedure of were performed. This simulation process created a sample of bootstrapped means for costs and QALYs with distributions for each. The means and other parametric statistics were then calculated for the bootstrap distribution. Bootstrap estimates of the difference in costs and QALYs to pay between the experimental and control arms were used to populate the cost-effectiveness plane and cost-effectiveness acceptability curve (CEAC).^29^ A cost-effectiveness plane and CEAC are ways of demonstrating the probability of an intervention’s cost-effectiveness across a range of different values. The former in the form of a scatter plot and the latter with these points expressed on an X and Y axis.^30^ The results of the bootstrapping were used to estimate the probability of each management strategy being considered cost-effective at different societal willingness to pay for a QALY. For example, the NICE cost-effectiveness threshold of £30 000 per QALY.^25^ In terms of deterministic sensitivity analysis, QALYs were recalculated using both the utility values generated from the HUI3 and GUI quality of life tools to see if this changed the economic conclusions. The inclusion of patient time and travel costs were included to assess the impact potential conclusions.

### Handling missing data

With respect to costs, cost CRF data were reported as missing under two circumstances, first where either the section reporting medications taken or the procedures undertaken were completely unreported (no values were given, positive or negative in either section). Second, costs were also reported as missing if the total costs for healthcare resources reported on the CRF was 0 and the response to the question ‘Has participant completed the TAGS Participant Questionnaire?’ was no. With respect to the estimation of QALYs for this base case analysis, those who had four of the seven data points were included for analysis. First, to account for the missing data points, the assumption that the previous utility remained stable was assumed. This meant the weighted average of the two utility scores around the missing values was used to calculate the missing data. After this, multiple imputation was used to estimate missing utility values for QALY scores.^31^

## Results

### Completeness of data

Data completion in this trial was generally very high as can be seen in online supplemental table A6. No patterns were observed in the missing QALY data, so the data were imputed randomly.

### Cost estimates

The unadjusted costs are summarised in table 1. The mean cost per patient in the trabeculectomy arm was £3826 (95% CI 3600 to 4050) and in the medical arm was £1685 (95% CI 1490 to 1880), there was a significant difference in costs between the two arms £2141 (p=<0.01).

**Table 1:** Total unadjusted costs in each arm in first and second 12 months of trial follow-up

Total cost	Trabeculectomy		Medical management	
	Mean	SD	Mean	SD
Total cost to the NHS over 24 months (£)	3826	1648	1685	1401
Total cost to the NHS between baseline and 12 months (£)	3157	1299	1067	1299
Total cost to the NHS between 12 and 24 months (£)	669	977	618	632

NHS, National Health Service.

### QALY estimates

The results for the QALY scores produced by the three quality of life instruments are summarised in table 2. For the EQ-5D-5L, there was evidence of slightly higher QALYs at 24 months for the trabeculectomy compared with the medical arm in the unadjusted mean difference.

**Table 2:** Utility values, QALYs for each utility measure by study arm along with differences in QALYs at 24 months

Treatment	Trabeculectomy	Medical management
	Mean (SD) (n)	Mean (SD) (n)
**Effectiveness**		
EQ-5D-5L baseline (n=444)	0.84 (0.18) (222)	0.84 (0.18) (222)
EQ-5D-5L 1 month (n=397)	0.84 (0.18) (194)	0.81 (0.20) (203)
EQ-5D-5L 3 months (n=365)	0.84 (0.17) (186)	0.81 (0.20) (179)
EQ-5D-5L 6 months (n=381)	0.85 (0.18) (186)	0.82 (0.20) (195)
EQ-5D-5L 12 months (n=420)	0.84 (0.18) (211)	0.82 (0.16) (209)
EQ-5D-5L 18 months (n=365)	0.83 (0.19) (181)	0.79 (0.22) (184)
EQ-5D-5L 24 months (n=409)	0.81 (0.18) (203)	0.80 (0.19) (206)
**Complete QALYs over 24 months using EQ-5D-5L (n=290)**	**1.65 (0.24) (144**)	**1.59 (0.28) (146**)
HUI3 baseline (n=428)	0.81 (0.20) (214)	0.80 (0.20) (214)
HUI3 1 month (n=377)	0.79 (0.23) (184)	0.79 (0.23) (193)
HUI3 3 months (n=359)	0.80 (0.22) (180)	0.78 (0.22) (179)
HUI3 6 months (n=362)	0.81 (0.22) (180)	0.78 (0.22) (182)
HUI3 12 months (n=400)	0.83 (0.19) (204)	0.80 (0.20) (196)
HUI3 18 months (n=343)	0.80 (0.21) (169)	0.75 (0.26) (174)
HUI3 24 months (n=391)	0.79 (0.23) (198)	0.75 (0.25) (193)
**Complete QALYs over 24 months using HUI3 (240)**	**1.61 (0.30) (123**)	**1.54 (0.36) (117**)
GUI baseline (n=441)	0.88 (0.13) (219)	0.86 (0.13) (222)
GUI 1 month (n=399)	0.86 (0.14) (194)	0.85 (0.16) (205)
GUI 3 months (n=377)	0.85 (0.13) (187)	0.84 (0.16) (190)
GUI 6 months (n=377)	0.84 (0.16) (186)	0.85 (0.14) (191)
GUI 12 months (n=413)	0.86 (0.14) (209)	0.86 (0.14) (204)
GUI 18 months (n=365)	0.85 (0.14) (181)	0.83 (0.16) (184)
GUI 24 months (n=407)	0.85 (0.15) (205)	0.83 (0.18) (202)
**Complete QALYs over 24 months using GUI (n=293)**	**1.67 (0.20) (144**)	**1.64 (0.24) (149**)

GUI, Glaucoma Utility Index; HUI3, Health Utilities Index Mark 3; QALY, Quality-Adjusted Life-Year.

### Economic evaluation

**Figure 1:**
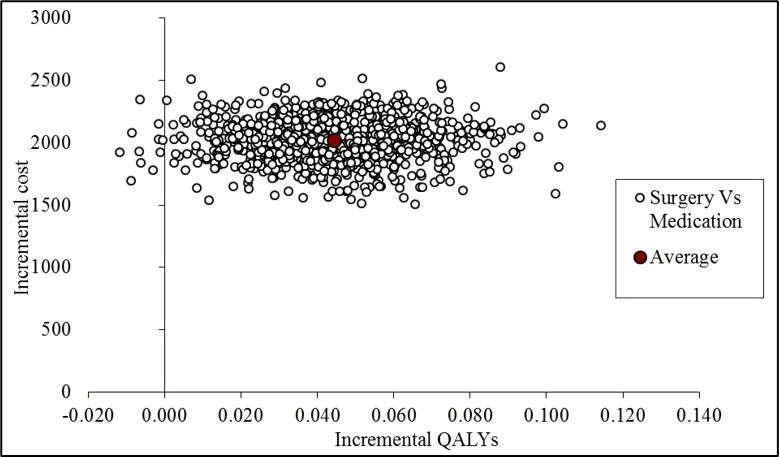
Cost-effectiveness plane for trabeculectomy versus medical management—adjusted bootstrapped replications for CUA for EQ-5D-5L results. CUA, cost-utility analysis. QALY, quality-adjusted life year.

**Figure 2:**
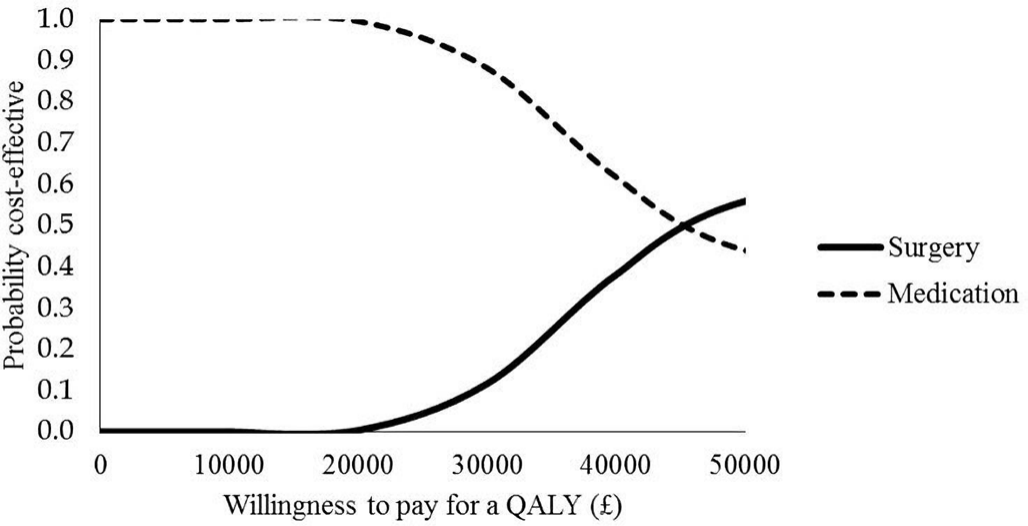
Cost-effectiveness curves for the trabeculectomy and medical management using the results from the EQ-5D-5L using the multiple imputation data. QALY, quality-adjusted life year.

The results of the CUA are presented in table 3, figures 1 and 2. The results of the SUREG display that the trabeculectomy arm is on average more costly and more effective than medical management. Using the larger multiple imputed data set, the incremental cost per QALY at 2 years is £45 456. The effectiveness plane (figure 1) demonstrates the difference in costs and QALYs for surgery compared with medical management are almost entirely in the quadrant which represents greater effect at greater cost for surgery compared with medical management. The probability of surgery being cost-effective at a £20 000, £30 000 and £50 000 QALY threshold is 0%, 12% and 56% respectively. The CEAC in figure 2 shows that at 2 years follow-up, surgery is unlikely to be considered cost-effective over the range of values that society might be willing to pay for a QALY.

**Table 3:** Complete and multiple imputation EQ-5D-5L results

EQ-5D-5L data	Intervention	Unadjusted cost (£) (95% CI)	Adjusted* incremental cost (£) (95% CI)	Unadjusted† QALY (95% CI)	Adjusted incremental QALY (95% CI)	ICER (ΔCost/ ΔQALY) (£)	Probability of being cost-effective at different thresholds for a QALY			
							£0	£20 000	£30 000	£50 000
Complete case data (n=290)	Trabeculectomy (n=144)	3686 (3435 to 3937)	2089 (1762 to 2416)	1.65 (1.61 to 1.69)	0.03 (0.01 to 0.08)	64 303	0	0%	6%	35%
	Medical management (n=146)	1605 (1390 to 1820)		1.59 (1.55 to 1.64)			100%	100%	94%	65%
Imputation data (n=403)	Trabeculectomy (n=199)	3622 (3372 to 3872)	2013 (1699 to 2327)	1.61 (1.57 to 1.65)	0.04 (−0.01 to 0.08)	45 456	0	0%	12%	56%
	Medical management (n=204)	1605 (1409 to 1801)		1.56 (1.52 to 1.60)			100%	100%	88%	44%

*Adjusted results are based on the results of the SUREG.

†Unadjusted results are based on the trial data.

ICER, Incremental Cost-Effectiveness Ratio; QALY, Quality-Adjusted Life-Year.

The results of the cost-effectiveness analysis based on QALYs derived from responses to the HUI3 and GUI do not change the conclusion of the analysis (see online supplemental table A7, figures A1, A2 and table A8, figures A3, A4). In both instances, medication was more likely to be cost-effective at the 24-month follow-up, with the surgical arm displaying a small amount of extra effect for each instrument The inclusion of participant costs (which is shown in online supplemental table A9 and figures A5, A6) also did not change the overall conclusions of the study.

## Discussion

Over the 24-month follow-up of the trial, surgical treatment was more costly and more effective than medical management. The results of the stochastic analysis suggest that any increase in QALYs over 24 months follow-up is unlikely to be sufficient to compensate for the increased costs of the surgery. The principal driver of this result was the higher surgical and outpatient costs in the surgical arm.

### Comparison with other glaucoma studies

There have been no previous studies comparing cost-effectiveness between medications and surgery as a primary treatment for advanced glaucoma. There have been prior studies which have estimated the cost-effectiveness of glaucoma treatments in different populations. For example, in the LIGHT study selective laser trabeculectomy (SLT) was compared with medical management of ocular hypertension and early glaucoma. The study found SLT produced similar clinical results at a lower cost than medication.^32^Stein *et al* 33 also compared medications with laser trabeculoplasty (LTP) for the treatment of patients with newly diagnosed mild OAG using a Markov model with a 25-year time horizon. The results of which suggested medication was superior to LTP.^33^ These two studies differ from the TAGS as they focused on treatments for patients in the earlier stages of glaucoma. Only one study was identified that considered more severe glaucoma, Guedes *et al* 34 used a Markov model to identify the most cost-effective treatment strategy for each severity of glaucoma.^34^ The results of this study found that the surgery was cost-effective in participants who are less than 70 years old. Meaning this model found that those who will have a longer life expectancy appear to accrue benefit from the additional costs of trabeculectomy surgery over a longer period.

### Strengths and limitations

For the economic evaluation, one of the key limitations of the within-trial analysis was the limited follow-up in the trial (24 months). As OAG is a chronic, lifelong condition the full benefit (and costs) of each randomised intervention is unlikely to be captured within this time frame, as demonstrated by Guedes *et al*.^34^ Future research is underway to focus on the longitudinal costs and outcomes of the interventions over a longer time horizon.

The primary strength of this study was that it contains a large sample of homogeneous patients all of whom had advanced glaucoma. Another strength is the use of multiple measures HRQoL: the EQ-5D-5L, the HUI3 and a glaucoma specific measure, the GUI, allowing benefits to be captured by three different metrics. As the conclusion did not change dependent on metric then confidence in them is increased. The study also compared three different methods of assessing HRQoL in relation to eye disease. Interestingly, the EQ-5D-5L proved to be the most likely to detect a difference in HRQoL in glaucoma patients, though the difference between the all the metrics was small. This is despite the fact that the HUI3 had a specific question relating to participants’ vision and that the GUI was developed specifically for use in glaucoma patients and had a value set purposely developed for this trial population. The LIGHT trial also used the EQ-5D to measure quality of life. This trial reported a similar small difference in EQ-5D scores. This difference was smaller than that identified in TAGS.^32^ However, this aligns with the findings of Bozzani *et al*,^35^ which found the sensitivity of the EQ-5D to detect differences varied according to the stages of disease. Bozzani *et al* 35 went on to conclude that there is a need for future research to assess the measurement in terms of sensitivity and generalisabilty for measurement of eliciting HRQoL in patients with eye disease.^35^

Another consideration for the interpretation of the economic evaluation is the inclusion of the costs of managing disease in the non-index eye. When estimating total costs, both the costs for the index eye and non-index eye were included. This was done because the outcome measure of principle interest (QALYs) were not specific to the index eye but to the vision across both eyes. Also, the prognosis of one eye may affect decisions about the management of the other eye. The challenge that this represented for the analysis was that cost and benefits could be undervalued or overvalued if there was any imbalance in severity of disease in the non-index eye between the two arms. However, this was not the case in TAGS and that means that the impact of management of disease in the non-index eye was equally spread between the two arms and should not materially affect the marginal differences between the two randomised groups.

## Conclusion

This is the first study to evaluate accurately and prospectively the cost of treating newly diagnosed advanced glaucoma with the two currently standard treatment approaches. For treating those with advanced OAG both medical and surgical are viable treatment options in terms of HRQoL outcomes. At a 2-year time horizon, medication is the more cost-effective approach at managing glaucoma. Further research will follow the longitudinal benefits of surgical and medical intervention beyond the 2-year time horizon, as there is evidence from previous economic modelling studies that surgery could be considered cost-effective over a longer time horizon.

## Data Availability

Data are available upon reasonable request. Data will be available beginning 10 months and ending four years after publication of this paper. Data will be available for researchers who provide a methodologically sound scientific proposal, which has been approved by an ethics committee. Proof of the latter should be provided. Analyses should achieve the aims reported in the approved proposal. Requests for data sharing should be made to the corresponding author at ashleigh.kernohan@newcastle.ac.uk.
