# Supplementary material for: Cost-effectiveness of primary surgical versus primary medical management in the treatment of patients presenting with advanced glaucoma
Source: Br J Ophthalmol. 2022 Jul 26;107(10):1452–7. doi: 10.1136/bjo-2021-320887 (PMC10579172; doi:10.1136/bjo-2021-320887)
Supplement: Supplementary data [file bjo-2021-320887supp001.pdf]

Additional Tables for Within Trial Economic Evaluation Paper

Table A1: Unit costs for hospital appointments

Hospital Appointments				
**Item**	**£**	**Unit**	**Reference**	**Notes**
**Inpatient**	448	Per night	National Reference Costs 2017/2018	Ward costs per night
**Outpatient appointment**	105.09	Per appointment	National Reference Costs 2017/2018	Consultant led Ophthalmology outpatient costs

Table A2: Unit costs for glaucoma procedures

Ophthalmic Procedure	Source	HRG Code	Unit Cost	Comments
Trabeculectomy (Day Case)	National Reference Costs 2017/2018	BZ92B	1639.44	Very Major, Glaucoma or Iris Procedures, with CC Score 0-1
Trabeculectomy (Inpatient)	National Reference Costs 2017/2018	BZ92B	2184.07	Very Major, Glaucoma or Iris Procedures, with CC Score 0-1
Massage	National Reference Costs 2017/2018	BZ24G	143.29	Minor, Glaucoma or Iris Procedures
Adjustment / suturelysis / releasable release	National Reference Costs 2017/2018	BZ95	143.29	Minor, Glaucoma or Iris Procedures
5-FU injection	National Reference Costs 2017/2018	BZ95Z	149.69	Minor, Glaucoma or Iris Procedures + Unit Costs of Fluorouracil
Steroid Injections	National Reference Costs 2017/2018	BZ95Z	150.16	Minor, Glaucoma or Iris Procedure + Unit Prednisolone
Needling + 5-FU injection	National Reference Costs 2017/2018	BZ94B	149.69	Minor, Glaucoma or Iris Procedures + Unit Costs of Fluorouracil
Revision of bleb NEC	National Reference Costs 2017/2018	BZ91B	1347.56	Complex, Glaucoma or Iris Procedures, with CC Score 0-1
Reformation of anterior chamber of eye	National Reference Costs 2017/2018	BZ94B	127.90	Intermediate, Glaucoma or Iris Procedures, with CC Score 0
Phaco and IOL	National Reference Costs 2017/2018	BZ32B	878.61	Intermediate, Cataract or Lens Procedures, with CC Score 0-1

Table A3: Unit costs for glaucoma medications

Medication	Dose	Administration	Unit cost (£)	Source	Comments
**Prostaglandin Analogues (PA)**					

Medication	Dose	Administration	Unit cost (£)	Source	Comments
Saflutan	15 micrograms/ml	Single dose unit eye drop	12.20	BNF 2018 (Online)	Overall costs per box
Latanoprost	50 micrograms/ml	Eye drop	1.53	BNF 2018 (Online)	Overall costs per 2.5ml bottle
Bimatoprost	300 micrograms/ml	Eye drop	10.30	BNF 2018 (Online)	Overall costs per 3ml bottle,
Travoprost	40 micrograms/ml	Eye drop	7.27	BNF 2018 (Online)	Overall costs per 2.5ml
**Average Cost PA**			**7.83**		
**Carbonic Anhydrase Inhibitors (CAI)**					
Brinzolamide	10mg/ml	Eye drop	1.89	BNF 2018 (Online)	Overall costs per 5ml bottle
Dorzolamide	20mg/ml	Eye drop	1.55	BNF 2018 (Online)	Overall costs per 5ml bottle
**Average Cost CAI**			**1.72**		
**Alpha-2 Adrenergic Agonists (AA)**					
Brimonidine	0.2%	Eye drop	1.13	BNF 2018 (Online)	Overall costs per 5ml bottle
Iopidine	5mg/ml	Eye drop	10.88	BNF 2018 (Online)	Overall costs per 5ml bottle
**Average Cost of AA**			**6.01**		
**Beta Blockers (BB)**					
Timolol	0.25%	Eye drop	0.78	BNF 2018 (Online)	Overall costs per 5ml bottle
Betoptic	0.5%	Eye drop	1.90	BNF 2018 (Online)	Overall costs per 5ml bottle
**Average Cost of BB**			**1.34**		
**Parasympathetic Drops (Para)**					
Pilocarpine hydrochloride	1%	Eye drop	20.78	BNF 2018 (Online)	Overall costs per 10ml bottle
**Average Cost of Para**			**20.78**		
**Oral Glaucoma Medications**					

Medication	Dose	Administration	Unit cost (£)	Source	Comments
Acetazolamide	250mg	Tablet	**16.66**	BNF 2018 (Online)	Overall cost per box (30)
		**Combination Glaucoma Medications (comb)**			
Bimatoprost with timolol (AZARGA)	10mg/ml	Eye drops	11.05	BNF 2018 (Online)	Overall cost per 5ml bottle
Bimatoprost with timolol (Ganfort)	5mg/m	Eye drops	14.16	BNF 2018 (Online)	Overall cost per 3ml bottle
Brinzolamide with brimonidine (Simbrinza)	2mg/ml	Eye drops	9.23	BNF 2018 (Online)	Overall cost per 5ml bottle
Dorzolamide with timolol (Cosopt)	5mg/ml	Eye drops	1.50	BNF 2018 (Online)	Overall cost per 5ml bottle
Dorzolamide with timolol unit dose (Cosopt)	5mg/ml	Single dose unit eye drop	28.59	BNF 2018 (Online)	Unit Dose,60 doses
Dorzolamide with timolol (Eylamdo)	5mg/ml	Eye drop	14.29	BNF 2018 (Online)	Overall cost per 5ml bottle
Travoprost with timolol (DuoTrav)	5mg/ml	Eye drop	13.95	BNF 2018 (Online)	Overall cost per 5ml bottle
**Average Cost of Comb**			**13.25**		
		**Non-Glaucoma Medications**			
**Steroid Eye Drops**					
Dexamethasone with hypromellose, neomycin and polymyxin B (Maxitrol)	Dexamethasone 1 mg per 1 gram Neomycin (as Neomycin sulfate) 3500 unit per 1 gram Polymyxin B sulfate 6000 unit per 1 gram	Eye drop	1.68	BNF 2018 (Online)	Overall cost per 5ml bottle
Betamethasone sodium phosphate	1 mg per 1 ml	Eye drop	2.32	BNF 2018 (Online)	Overall cost per 5ml bottle
Dexamethasone (Maxidex)	1 mg per 1 ml	Eye drop	1.42	BNF 2018 (Online)	Overall cost per 5ml bottle
Prednisolone (Pred Forte)	10 mg per 1 ml	Eye drop	1.82	BNF 2018 (Online)	Overall cost per 5ml bottle

Medication	Dose	Administration	Unit cost (£)	Source	Comments
**Antibiotic Eye Drops**					
Azithromycin (azyter)	15 mg per 1 gram	Eye drop	1.17	BNF 2018 (Online)	Unit Dose, 6 doses
Choloramphenicol	5 mg per 1 ml	Eye drop	1.14	BNF 2018 (Online)	Overall cost per 10ml bottle
Celluvisc Unit Dose	1%	Single dose unit eye drop	4.80	BNF 2018 (Online)	Unit dose, 30 doses
Hylo-Forte unit dose	0.2%	Single dose unit eye drop	5.60	BNF 2018 (Online)	Unit dose, 30 doses
Sodium hyaluronate (Vismed Multi)	0.18%	Eye drop	6.87	BNF 2018 (Online)	Overall cost per 10ml bottle
**Mydriatics**					
Cyclopentolate (Mydrilate)	5 mg per 1 ml	Eye drops	8.08	BNF 2018 (Online)	Overall Cost per 5ml bottle
Atropine	10 mg per 1 ml	Eye drops	15.10	BNF 2018 (Online)	Unit Dose, 20 doses
**NSAID**					
Bromfenac (Yellox)	900 microgra m per 1 ml	Eye drops	8.50	BNF 2018 (Online)	Overall cost per 5ml bottle
**Sympathomimetic**					
Phenylephrine	50 microgram per 1 ml	Eye drops	11.87	BNF 2018 (Online)	Unit Dose, 20 doses
**Average Cost of Non Glaucoma Drops**			7.27		

Table A4: Unit costs for community appointments

Item	Unit	£	Comments
			**GP**
GP visit at their practice	Per 9.22 minute appointment	37	**PSSRU 2018**
GP home visit	11.4 minute Per appointment	45.98	**PSSRU 2015 (most recent info)**<br>11.4 minutes (2015 Health and Social Care) x 2017 hourly rate (£242) (no travel costs)
Telephone triage with GP	Cost per call	8.10	**PSSRU 2017 (most recent info)**<br>15.5 minutes x2015 hourly rate (£67) (not including travel)
			**Nurse**
Practice Nurse consultation	15.5 minutes per consultation	10.84	**PSSRU 2018**<br>15.5 minutes (length of appointment Unit Costs 2015) x 2017 hourly rate (£42)
District Nurse	25 minutes per consultation	17.29	**PSSRU 2017**<br>15.5 minutes x2015 hourly rate (£67) (not including travel)
			**Optician**
Optometrist in practice	Per examination	21.31	**Department of Health and Social Care**<br>Eye exam fee (As participants have glaucoma all will be entitled to NHS eye examinations)
Optometrist at home	Per examination	58.87	**Department of Health and Social Care**<br>Eye exam fee + domiciliary fee (As participants have glaucoma all will be entitled to NHS eye examinations)

Table A5. Costs for different resources in each arm.

Resource	Total cost (£) in the Trabeculectomy			Total cost (£) in the Medical management		
	**Mean**	**Median**	**Standard Deviation**	**Mean**	**Median**	**Standard Deviation**
GP Surgery consultations	26	0	103	13	0	34
GP Home consultations	3	0	20	2	0	18
GP telephone consultations	22	0	63	32	0	97
Practice Nurse consultations	26	0	49	45	0	128
District Nurse consultations	10	0	81	8	0	33
Optometrist consultations	29	21	38	21	21	25
Ophthalmology consultations	1313	1129	615	593	407	524
Other Consultations	41	0	246	69	0	311
Nights in Hospital	28	0	166	10	0	78
Releasable release	101	0	160	17	0	59
Ocular Massage	97	0	192	5	0	30
Trabeculectomy	1821	1639	850	394	0	800
5-Fluorouracil injection	30	0	95	8	0	37
Steroid injection	18	0	59	6	0	44
Needling + 5-Fluorouracil injection	37	0	99	10	0	54
Bleb resuturing	20	0	160	7	0	94
Anterior Chamber reformation	3	0	20	1	0	9
Bleb revision	45	0	242	7	0	94
Phacoemulsification	63	0	309	81	0	374
Prostaglandin Analogues	181	178	141	286	320	120
Carbonic Anhydrase Inhibitor	7	0	15	18	0	25

Beta Blockers	9	0	16	19	10	22
Alpha Agonists	5	0	19	15	0	50
Pilocarpine	0	0	5	1	0	21
Combinations	4	0	22	1	0	10
Diamox	1	0	10	1	0	18
Others	0	0	4	0	0	0

Table A6. Data completion in each arm

**Data response rates**	**Trial arm**	
	**Trabeculectomy %(n)**	**Medical management %(n)**
**Case Report Form**		
4-months	99% (226)	99% (223)
12-months	99% (226)	100% (226)
24-months	96% (217)	97% (219)
**EQ-5D-5L**		
Baseline	98% (222)	98% (222)
1-month	85% (194)	90% (203)
3-months	82% (186)	79% (179)
6-months	82% (186)	86% (195)
12-months	93% (211)	92% (209)
18-months	80% (181)	81% (184)
24-months	91% (206)	90% (203)
**Resource Use Questionnaire**		
4-months	93% (210)	96% (216)
12-months	94% (213)	92% (208)
24-months	92% (208)	90% (204)
**Time and Travel Questionnaire**	68% (154)	65% (148)

Table A7: Complete and MI HUI 3 Cost Utility Analysis Results

HUI 3 Data	Intervention	Unadjusted Cost (£)	Adjusted Incremental Cost (£)	Unadjusted QALY	Adjusted Incremental QALY	ICER (ΔCost/ ΔQALY) (£)	Probability cost-effective at threshold £0	£20,000	£30,000	£50,000
Complete case data (n=240)	Surgery	3819	2129	1.61	0.06	33,758	0%	9%	38%	76%
	Medication	1691		1.54			100%	91%	62%	24%
Imputation data (n=382)	Surgery	3688	2040	1.54	0.06	36,130	0%	4%	32%	72%
	Medication	1640		1.48			100%	96%	68%	28%

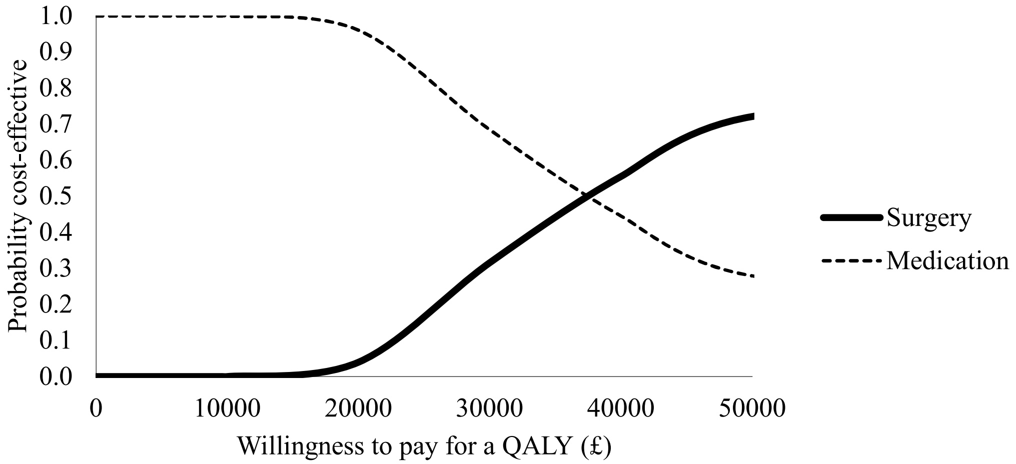

Figure A1: Cost effectiveness curves for the surgical and medical arms using the results from the imputed HUI3 sample

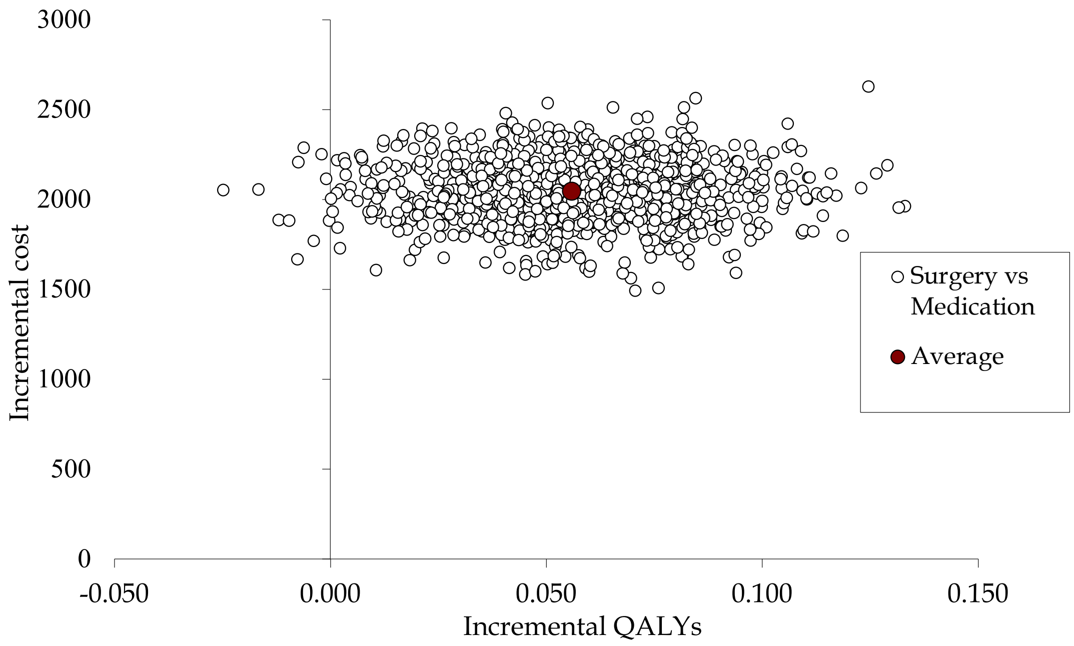

Figure A2: Cost effectiveness curves for the surgical and medical arms using the results from the imputed HUI3 sample

Table A8: Complete and MI GUI Cost Utility Results

GUI Data	Intervention	Unadjusted Cost (£)	Adjusted Incremental Cost (£)	Unadjusted QALY	Adjusted Incremental QALY	ICER (ΔCost/ΔQALY) (£)	Probability cost-effective at threshold			
							£0	£20,000	£30,000	£50,000
Complete case data (n=293)	Surgery	3683	2138	1.67	0.01*	111,117	0%	9%	38%	76%
	Medication	1541		1.64			100%	91%	62%	24%
Imputation data (n=398)	Surgery	3617	1995	1.64	0.00	350,149	0%	0%	0%	0%
	Medication	1615		1.62			100%	100%	100%	100%

*Adjusted difference is in favour of medication in this instance not surgery.

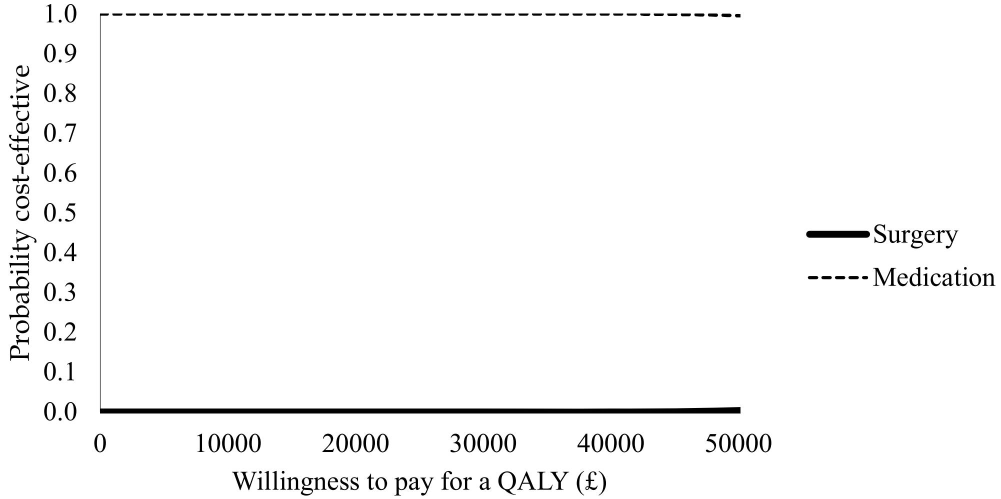

Figure A3: Cost effectiveness curves for the surgical and medical arms using the results from the imputed GUI sample

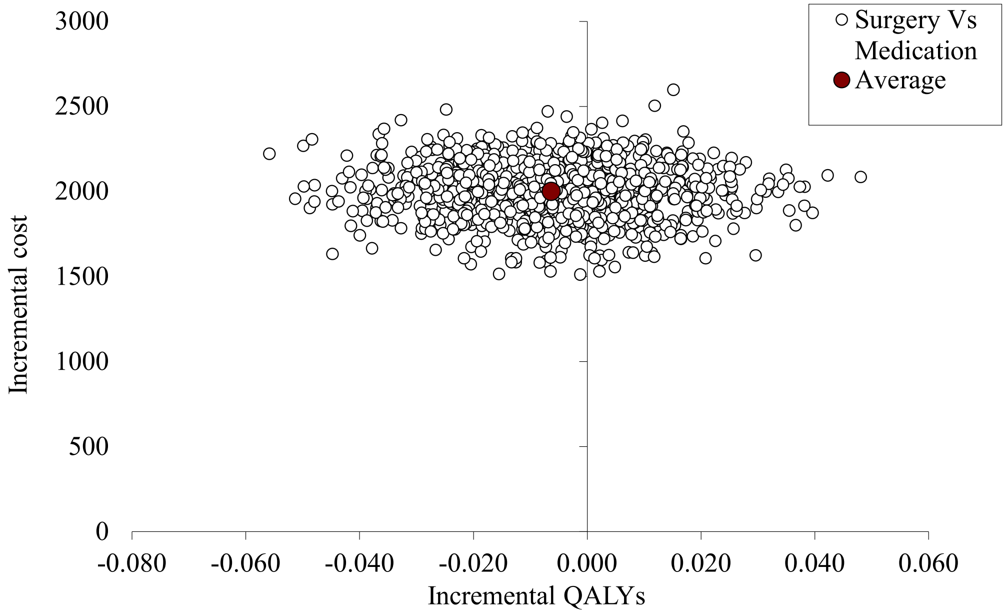

Figure A4: Cost effectiveness curves for the surgical and medical arms using the results from the imputed GUI sample

Table A9: EQ-5D-5L Data with patient time and travel costs included Cost Utility Results

EQ-5D-5L Data with patient time and travel costs included	Intervention	Unadjusted Cost with TT (£)	Adjusted Incremental Cost (£)	Unadjusted QALY	Adjusted Incremental QALY	ICER (ΔCost/ ΔQALY) (£)	Probability cost-effective at threshold			
							£0	£20,000	£30,000	£50,000
Complete case data (n=290)	Surgery	4453	2412	1.65	0.03	75,347	0%	1%	2%	25%
	Medication	2046		1.59			100%	99%	98%	75%
Imputation data (n=403)	Surgery	4419	2359	1.61	0.04	54,197	0%	1%	5%	42%
	Medication	2052		1.56			100%	99%	95%	58%

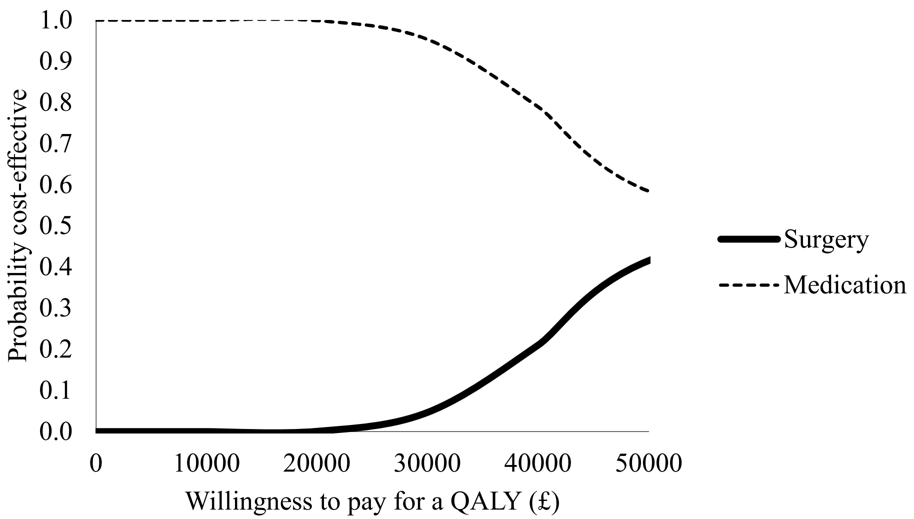

Figure A5: Cost effectiveness curves for the surgical and medical arms using the results from the imputed ED-5D-5L using the time and travel data

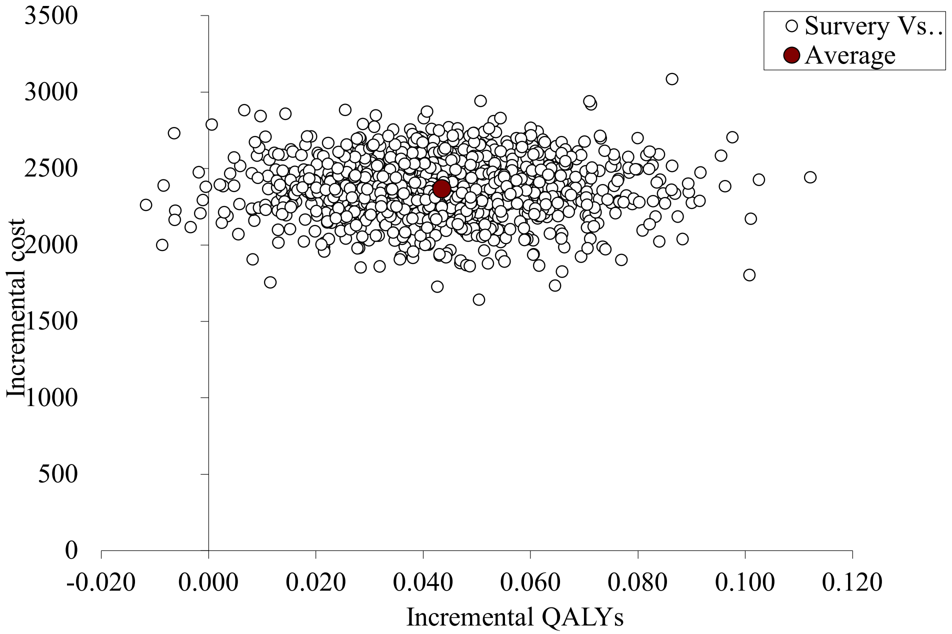

Figure A6: Cost-effectiveness plane for adjusted bootstrapped replications for Cost-utility analysis from the imputed ED-5D-5L results with time and travel costs
